# Supplementary material for: Doppelganger-based training: Imitating our virtual self to accelerate interpersonal skills learning
Source: PLoS One. 2021 Feb 10;16(2):e0245960. doi: 10.1371/journal.pone.0245960 (PMC7875421; doi:10.1371/journal.pone.0245960)
Supplement: Attachment — Submitted filename: Response to Reviewers.docx [file pone.0245960.s002.docx]

Internef Building

CH-1015 Lausanne

Dear Professor Friedman, dear Reviewers,

In the following pages of this letter, you will find the comments made by the reviewers in bold followed by a description of the revisions we have made.

We wish to thank you for the comments. They have greatly improved the present article.

Best regards,

Dr. Emmanuelle P. Kleinlogel

Researcher and Lecturer

Faculty of Business and Economics

University of Lausanne

**Reviewer #1**

**The authors effectively addressed the concerns of the reviewers. In particular, my minor concerns were addressed and those of the other reviewer were, in my opinion, addressed with appropriate references to the literature and caution to the readers to not jump to potentially false conclusions.**

Thank you for your positive feedback.

**Reviewer #2**

**Why was there not a control that did not include virtual human training? I feel as though there should be more discussion on if just repeatedly speaking in this way tends to lead to equivalent or better. You may be able to say that doppelgangers are better to use than Unknown Agents but without a control how can you tell this is an effective means of training?**

We thank you for this comment. We agree that this point needs to be clarified. We investigate the extent to which new technologies – VR – can contribute to improve trainees’ learning process. Effective training is composed of four phases (IDPF model of training), namely information, demonstration, practice, and feedback. Each phase is crucial during the learning process of interpersonal skills (see Bedwell, Fiore, & Salas, 2014) and it is important to investigate the best way of delivering to trainees each phase. In our research, we focused on the demonstration phase of the IDPF model of training. Drawing on past research on the use of doppelgangers as role models, we further documented whether this new way of learning through VR-based demonstration can help trainees improving their interpersonal skills in the specific context of public speaking. To assess the effect of doppelgangers as role models on trainees’ learning, in our study we compare two different demonstration conditions within VR, i.e. an experimental condition in which participants watched their doppelganger as a role model and a comparison condition in which participants watched a virtual person that do not resemble them.

We did not add a third condition that does not include a role model because our work focuses on the demonstration phase of training, hence involving a virtual person as a trainer. Moreover, adding another condition without virtual agents or without VR would have made the comparison difficult because then more than just one feature of the setting would have changed and thus introduced potential confounding variables. In the abstract, we presented the main goal of our work as followed (lines 27-29):

“In the present study, using the technology of immersive virtual reality we investigate the extent to which the use of doppelgangers as role models can boost trainees’ interpersonal skills development.”

We agree that this sentence can bring confusion to the readers regarding our research question. Hence, based on your comment, we decided to clarify this point as followed (lines 27-32):

“In the present study, given that virtual reality is emerging as a valuable training setting, we compare two different demonstration conditions within virtual reality by investigating the extent to which the use of doppelgangers as role models can boost trainees’ interpersonal skills development as compared to a role model that does not resemble the trainees.”

Furthermore, on pages 3-4, we inform the readers that we focus on the demonstration phase, hence involving trainees who observe a trainer – a role model – performing the desired behaviours. Nonetheless, similar to the abstract and based on your comment, we decided to be more precise to avoid confusion about the goal of our research by explaining on lines 73-77 that:

“In the present research, given that VR is emerging as a valuable training setting, our goal is to compare two different demonstration conditions within VR. Specifically, we investigate the effectiveness of using a doppelganger as a role model in the development of interpersonal skills as compared to a role model that does not resemble the trainees.”

**When it comes to enhancing skills in public speaking there was a lack of prior work provided. The authors heavily relied on the work by Aymerich-Franch and Bailenson but this work focuses on social-anxiety and not improving interpersonal communication skills (except one could argue stress management but that does not seem to be the focus for this manuscript). I would suggest some background on non-verbal gesture or other public speaking skills training be discussed more in depth.**

Thank you for this comment. Indeed, in our paper we heavily rely on the work by Aymerich-Franch and Bailenson. Their work is central in the field of public speaking VR-based training because, to our knowledge, it is the first study that investigated the effect of doppelgangers as role models on trainees’ learning in the demonstration phase.

Previous research on public speaking has mainly investigated the effectiveness of VR-based training during the practice phase (e.g., Anderson et al., 2005; Gorini & Riva, 2008; Harris et al., 2002; Lindner et al., 2019; Stupar-Rutenfrans et al., 2019). Furthermore, these studies mainly focused on the fear of public speaking as an outcome. Hence, there is a lack of research on public speaking skills development per se involving the technology of VR.

In our paper, we wrote that traditionally, the demonstration phase involved the use of videos, however nowadays new technologies allow new ways of designing training (lines 62-66):

“In the present research, we focus on the effect of role models for learning and training. Role models are part of the demonstration phase in which trainees observe individuals performing the desired behaviours so that they can learn through behavioural mimicry [4, 5]. Traditionally, demonstration involves the use of videos allowing trainees to watch examples of desired and undesired behaviours [2]. With the emergence of new technologies such as immersive Virtual Reality (VR) and the use of virtual humans for training, there are new possibilities available for training [6].”

We agree that it would be informative for the readers to discuss in more detail past research. We now briefly present the current state of research on lines 66-72:

“Past research has documented VR as an effective tool in interpersonal skills development and more specifically in public speaking training [7-11]. However, these studies have focused on the practice phase of training by showing positive outcomes after trainees’ participation to VR-based training sessions. Furthermore, these studies have mainly assessed VR as a tool to reduce public speaking anxiety, hence neglecting other interpersonal skills. To date, there is thus a lack of research on public speaking development per se involving the technology of VR.”

**321 -324 "As expected, findings revealed that the use of a doppelganger led to better performance than the use of a same gender avatar role model. However, we observed this effect only among male participants that were relatively low in self-efficacy, as measured following the VR training." The first sentence here is a very strong statement that I don't believe can be stated here considering the small size of the group that once divided up showed the result. I would suggest weakening the statement or leading with the second statement and stating that this provides some evidence towards your hypothesis.**

We agree that, as mentioned on lines 384-386, “Our findings should be interpreted with caution due to our relatively small sample size […]” and hence these statements are too strong. Accordingly, as suggested, we toned down the statements related to our findings. It now reads on lines 333-340:

“Results went in the expected direction by showing that the use of a doppelganger helped improving performance as compared to the use of a same gender avatar role model. However, findings suggested this effect to be specific to male participants that were relatively low in self-efficacy, as measured following the VR training. These results provide some evidence towards our expectation that individuals relatively low in self-efficacy would benefit the most from doppelganger-based training than individuals with relatively higher levels of self-efficacy.”

**326-329 "We argue that, by watching their virtual self delivering the charismatic speech, participants relatively low in self-efficacy had a greater motivation to put effort into the task and to persevere than those who watched an unknown avatar performing the same charismatic speech." This effect could also be seen as a result of participants with lower self-esteem feeling better about the task after practicing the first time. It would have been interesting to see self-efficacy measured before and after the first public speech as it is unknown if self-efficacy ratings were affected by performing. (Potentially increasing for those that had previous anxiety or the other way around).**

Thank you for this comment. Indeed, it would be interesting to capture variation of participants’ level of self-efficacy throughout the training. Hence, it would allow assessing whether the public speaking task leads to increase or weaken participants’ level of self-efficacy depending on how they perform, as well as assessing how this variation affects their subsequent performance. We added this idea of future research on lines 404-407. It reads:

“Furthermore, it would be interesting to study whether trainees’ level of self-efficacy varies throughout the training and if yes, to assess how and the extent to which this variation is affected by the performance of trainees as well as the extent to which it affects their subsequent performance.”
